# Changes in Language Style and Topics in an Online Eating Disorder Community at the Beginning of the COVID-19 Pandemic: Observational Study

**DOI:** 10.2196/28346

**Published:** 2021-07-08

**Authors:** Johannes Feldhege, Markus Moessner, Markus Wolf, Stephanie Bauer

**Affiliations:** 1 Center for Psychotherapy Research Heidelberg University Hospital Heidelberg Germany; 2 Department of Psychology University of Zurich Zurich Switzerland

**Keywords:** COVID-19, eating disorders, online eating disorder community, language, mental health, social media, LIWC, Linguistic Inquiry and Word Count, Reddit, topic modeling

## Abstract

**Background:**

COVID-19 has affected individuals with lived experience of eating disorders (EDs), with many reporting higher psychological distress, higher prevalence of ED symptoms, and compensatory behaviors. The COVID-19 pandemic and the health and safety measures taken to contain its spread also disrupted routines and reduced access to familiar coping mechanisms, social support networks, and health care services. Social media and the ED communities on social media platforms have been an important source of support for individuals with EDs in the past. So far, it is unknown how discussions in online ED communities changed as offline support networks were disrupted and people spent more time at home in the first months of the COVID-19 pandemic.

**Objective:**

The aim of this study is to identify changes in language content and style in an online ED community during the initial onset of the COVID-19 pandemic.

**Methods:**

We extracted posts and their comments from the ED community on the social media website Reddit and concatenated them to comment threads. To analyze these threads, we applied top-down and bottom-up language analysis methods based on topic modeling with latent Dirichlet allocation and 13 indicators from the Linguistic Inquiry and Word Count program, respectively. Threads were split into prepandemic (before March 11, 2020) and midpandemic (after March 11, 2020) groups. Standardized mean differences were calculated to estimate change between pre- and midpandemic threads.

**Results:**

A total of 17,715 threads (n=8772, 49.5% prepandemic threads; n=8943, 50.5% midpandemic threads) were extracted from the ED community and analyzed. The final topic model contained 21 topics. CIs excluding zero were found for standardized mean differences of 15 topics and 9 Linguistic Inquiry and Word Count categories covering themes such as ED symptoms, mental health, treatment for EDs, cognitive processing, social life, and emotions.

**Conclusions:**

Although we observed a reduction in discussions about ED symptoms, an increase in mental health and treatment-related topics was observed at the same time. This points to a change in the focus of the ED community from promoting potentially harmful weight loss methods to bringing attention to mental health and treatments for EDs. These results together with heightened cognitive processing, increased social references, and reduced inhibition of negative emotions detected in discussions indicate a shift in the ED community toward a pro-recovery orientation.

## Introduction

COVID-19, caused by SARS-CoV-2, emerged in late 2019 in Wuhan, China and has since spread worldwide before being declared a global pandemic on March 11, 2020, by the World Health Organization. With the number of infections and deaths in the millions, the COVID-19 pandemic has already led to suffering for much of the world population. The pandemic has also contributed to elevated levels of anxiety, depression, and stress in the general population [1,2], with potentially greater effects on individuals with pre-existing mental disorders [3]. Quarantines, social distancing, lockdowns, and other public health measures taken to contain the spread of COVID-19 also have the potential for adverse effects on psychological well-being [4]. These measures were associated with increased depression, anxiety, and psychological distress in the general population [5], with indications for persisting effects after lockdowns were lifted [6]. During previous epidemics, experiences of quarantine led to long-term increases in depressive symptoms [7] and heightened anxiety symptoms, especially in those with a history of mental disorders [8]. Similarly, the COVID-19 pandemic and its associated public health measures impact individuals with lived experience of eating disorders (EDs) in numerous ways, affecting their symptomatology, social support, coping mechanisms, treatment, and engagement with media and the internet.

Individuals with EDs showed higher psychological distress including heightened fear and health anxiety since the beginning of the pandemic [9,10]. ED symptoms such as bingeing and food restriction have worsened [10-13], which has been linked to increased food insecurity, as opportunities to shop for food have been reduced and food shortages appeared in supermarkets [14]. Former patients with bulimia nervosa reported higher shape, weight, and eating concerns; body dissatisfaction; and drive for thinness [13]. Studies have also found a higher prevalence of compensatory behaviors such as purging, excessive exercise, or the abuse of laxative and diuretics during this time [9,10,12,13].

Health and safety measures due to COVID-19, such as quarantine, social distancing, or lockdowns disrupted the structure and routine of everyday life, as most people spent a lot more time at home than usual [15]. For many individuals with EDs, this has led to the loss of familiar coping mechanisms and increased rumination about food, weight, or exercise [11]. Similarly, social support has been impacted by health and safety measures that left many individuals isolated and cut off from their usual social support networks [10]. Additionally, access to health care services and ED treatment has been limited during this time [13,15,16]. Although many services transitioned to telehealth applications rather quickly, patients report that the quality of their treatment has declined during the pandemic [10]. At the same time, contacts to helplines and instant chats have increased compared to previous years with individuals contacting these services being more strongly affected by EDs, depression, or anxiety [17].

The COVID-19 pandemic also put topics such as food, weight gain, and physical exercise, which can be triggering for individuals with EDs, into the spotlight of traditional and social media [9,11]. This includes news coverage of food shortages and news reports about potential weight gain due to lower activity levels and increased time spent at home as well as a spread of online workout videos on social media [14,18].

For individuals with mental health issues, and especially for individuals with a lived experience of an ED, or those who are at risk for developing an ED, social media has been an important source of communication and social support from like-minded peers due to the shame and stigma associated with these conditions [19]. The expectation would be that social media became even more important for these individuals during the COVID-19 pandemic, a time when access to other sources of support is reduced and people have to spend more time at home. A mixed-methods study on the impact of the COVID-19 pandemic on individuals with lived experience of an ED in the United Kingdom reported that most participants are spending more time on the internet and on social media and that many felt that this had a negative effect on their ED symptoms [11]. One reason for the negative impact of time spent online might be that some participants visited potentially harmful online pro-ED communities, also called pro-ana (short for pro–anorexia nervosa) or pro-mia (short for pro–bulimia nervosa). These websites or social media channels are considered harmful because they often glorify EDs and encourage visitors to engage in pathological ED-related behaviors rather than supporting them to seek help or recovery from EDs [20,21]. Prominent features in pro-ED communities are “thinspiration” content, text or images that propagate a thin ideal, and weight loss “tips and tricks” [22,23]. Effects of visiting pro-ED communities are increases in dieting, body dissatisfaction, negative affect, drive for thinness, and disordered eating behaviors [21,24,25].

Although these communities can be harmful, they can also be a source of social support for individuals who feel that other users with lived experience of EDs can understand them better than real-life friends and family [26,27]. As a consequence, people with EDs, or those at-risk for developing an ED, could be reaching out to these communities during the COVID-19 pandemic to receive the social support they are lacking in their everyday life. A qualitative study on three ED communities on the social media website Reddit during the first months of the COVID-19 pandemic uncovered themes such as increased ED symptomatology, changes in daily routine, and treatment interruptions [28], which echo issues that were also found in survey studies.

Research on social media can complement, extend, and even offer some advantages over more traditional clinical research approaches. For example, on social media, a wide group of individuals is active including those at risk for EDs, those that have never been in treatment, or those that would not be reached by traditional surveys. Furthermore, the analysis of social media text is free from the biases introduced by experimental or interview situations. Social media research has contributed to our understanding of the social and psychological implications of disruptive events such as a global pandemic, for example, through tracking developments and interactions, and analyzing their temporal and geographical distributions [29]. Studying social media can also bring to light how an infodemic, that is, health-related misinformation, spreads [30]. A number of phenomena related to the COVID-19 pandemic have already been explored with data from social media websites. One study charted the course of COVID-19 symptoms based on topics and language styles extracted from firsthand accounts infected individuals had shared on Reddit [31]. Another study observed the impact of lockdowns on the language styles of Twitter users in Wuhan and Lombardy [32]. The researchers discovered changes in indicators for cognitive processing, uncertainty, and increased time spent at home.

In this study, we explored how social media activity in an online ED community develops during the first months of the COVID-19 pandemic. Although this time is certainly difficult for all individuals with mental health conditions, it posed a particular challenge for individuals with lived experience of EDs. Recent survey studies have shown not only that these individuals are at high risk of experiencing deteriorating symptomatology but also that they were particularly affected by the health and safety measures imposed to curb the spread of the virus and by the news reports on food shortages, weight gain, and home workouts. We sought to determine whether the aforementioned effects such as worsened ED symptomatology and anxiety and reduction of treatment services and social support are reflected in changes in one of the largest ED communities on the social media website Reddit. Due to lockdowns and quarantines, many individuals were spending more time on the internet and social media, potentially discovering ED communities for the first time or becoming more active in them. These communities differ from other mental health online communities in that they often contain both harmful and supportive elements. Therefore, it is vital to investigate the communities that individuals with lived experience of EDs can encounter on social media and how these communities changed as the COVID-19 pandemic began to develop. To the best of our knowledge, only one qualitative study has investigated online ED communities on the social network Reddit.com at the beginning of the COVID-19 pandemic [28]. In contrast to this study, we took a quantitative approach to one of the largest ED communities on Reddit by using two state-of-the-art quantitative text analysis methods. Such methods allow researchers to turn large samples of texts into quantitative representation and to estimate differences between chosen subsets. Specifically, we applied top-down and bottom-up automated language analysis methods to track verbal behavior during the early weeks of the pandemic. First, we extracted the major topics and estimated changes in their prevalence during this time. Second, by using a validated dictionary approach, we analyzed language styles in this community before and after the initial onset of the pandemic. These two analysis methods each provide distinct insights and at the same time complement each other to produce a richer understanding of the changes in an online ED community during the first months of the COVID-19 pandemic than either method on its own. Because our study focuses on the changes in the community as a whole and not on individual users, the unit of analysis for both methods is a discussion thread, which consists of an initial post and all comments made to this post. The aim of our study was to investigate possible changes in the content and language style of these threads at the beginning of the COVID-19 pandemic.

## Methods

### Study Design

We conducted an observational study in an online ED community to identify changes in content and language style in comment threads after COVID-19 became a global pandemic. We chose March 11, 2020, as the start date for the global pandemic, as it coincides with the declaration of COVID-19 as a global pandemic by the World Health Organization [33]. All data were categorized as *prepandemic* (before March 11) or *midpandemic* (after March 11) in a dichotomous variable *global pandemic status*. All topic and language style variables were *z* standardized, resulting in variables with a mean of 0 and a SD of 1, allowing for easier interpretation. Changes in topics and language styles from pre- to midpandemic threads were estimated by subtracting their midpandemic mean standardized prevalence from their prepandemic mean standardized prevalence. These standardized mean differences (SMDs) between pre- and midpandemic prevalences can be considered as analogues to effect sizes such as Cohen *d*. They were illustrated in a graph together with their 99% CIs. A significant change in the mean prevalence of a topic or language style can be observed if its CI does not include zero. A stricter level of confidence at 99% was chosen as even small differences can become significant in a large data set such as the one used in this study.

### Data Set

We collected data from a large ED community on the social media website Reddit. The community is not identified by name in this paper, as the users wish to remain anonymous. The community was founded in November 2017 after the largest ED community on Reddit at the time, *r/proed*, was shut down by the administrators of Reddit for violating its rules (for more information on *r/proed*, see [34]). Posts and comments in this community were accessed in regular time intervals from April 6 to May 20, 2020, through Reddit’s official application programming interface (API) using the R package redditoR. As the API limits the access to 1000 items at a time, posts and comments earlier than April 6 were not available through this approach. To gather earlier posts and comments and, thus, derive a prepandemic sample, we accessed earlier posts and comments in the ED community by users who had contributed at least one post or comment in the time period between April 6 and May 20. In total, 18,071 posts and 100,143 comments created by 6683 users between November 1, 2019, and May 20, 2020, were available for analysis. We concatenated the text of a post and the texts of all comments made to that post into a single thread, thereby combining texts from different users. This approach was deemed appropriate because the focus of our study was not on changes in individual users but rather on trends in topics and language stylistics in the community as a whole. In the following, threads are used as the unit of analysis.

### Data Preprocessing

We removed 195 posts and 7 comments made by self-identified bots from the data. The native language of posts and comments was determined using the R packages cld2 and cld3, and 22 comments in a language other than English were excluded from further analyses. The threads were prepared for the text analyses by removing HTML code and Unicode characters.

### Topic Modeling

We used topic modeling with latent Dirichlet allocation (LDA) [35] to discover latent topics in threads. LDA is a state-of-the-art unsupervised bottom-up text analysis method that has previously provided intelligible topics for text corpora from Reddit communities for depression [36] and EDs [34]. It can be applied on large text corpora without manual coding and little input by the researchers. Two additional text preprocessing steps were performed to prepare the threads for topic modeling. First, the removal of numbers, punctuation marks, and stop words, which are common words with little meaning, such as *is* or *this*. For this, a list of stop words created by the Snowball stemmer project and included in the R package tm was used. The second preprocessing step was reducing words to their word stem. The R package stm was used to estimate a structural topic model of the preprocessed threads. The variable *global pandemic status* was included as a covariate in the model, allowing the prevalence of topics in threads to vary according to whether they were started before or after the declaration of COVID-19 as a pandemic. An initial search for the appropriate number of topics, *K*, for the corpus was conducted with topic models with different values of *K*=3, 6, 9, 12...30. In this first step, the models were evaluated using the indicators exclusivity and semantic coherence to narrow down the number of topics [37,38]. In a second step, a number of models with *K*=9 to 21 topics were estimated. For these models, we set the number of runs to 50 for each K, the number of expectation-maximization iterations to a maximum of 200, and all other parameters to default values. From these candidate models, a final model with *K*=21 topics was chosen based on inspection of semantic coherence and exclusivity and manual evaluation of interpretability of its topics. The exclusivity and semantic coherence for models in both steps are displayed in Figures S1 and S2 in Multimedia Appendix 1. Topics were manually annotated with a topic label by the main author (JF), and topic labels were reviewed by the other authors (MM, MW, and SB). Labels were chosen on the basis of the 15 most characteristic words and 20 most characteristic texts for each topic. The Results section shows the manually chosen topic labels and 7 characteristic words as measured by the FREX metric, which balances how frequent and how exclusive to one topic a word is [37].

### Language Style Analysis

Language style in threads was assessed with a set of 13 indicators that have been shown to be associated with ED-related social online activities [39]. These indicators cover behavioral, affective, social, and cognitive dimensions of language use (see the Results section for the names and exemplary words for the indicators). To assess the frequency of these indicators, we used the Linguistic Inquiry and Word Count (LIWC) text analysis program [40]. LIWC is based on a word count algorithm that searches each text unit for words that are assigned to prespecified language categories in its internal dictionary. Words in a given text are matched to these categories and counted to determine the frequency of each category in the text. We also included the relative frequencies of question marks and exclamation marks provided by LIWC, as their use can be an indicator for complexity reduction in texts [39]. We excluded 161 (0.91%) threads from the analyses because less than 70% of their words were captured by the LIWC 2015 dictionary to prevent unreliable analyses (eg, short text units or due to misspellings or typing errors).

## Results

The final sample used in the analysis consisted of 17,715 threads (n=8772, 49.5% prepandemic threads; n=8943, 50.5% midpandemic threads). Descriptive statistics of the threads are listed in Table 1. An average thread contained 4 (SD 7.55) comments, was populated by 4.02 (SD 5.19) users, and consisted of 258.41 (SD 390.18) words. Of the 6554 users that participated in the final sample of threads, 2595 (39.59% of all users) participated in both pre- and midpandemic threads. These users participated in more threads per day in the midpandemic period (mean 0.12, SD 0.25) than in the prepandemic period (mean 0.08, SD 0.15; *t*_2594_=–9.18; *P*<.001). There were 3215 (49.05%) users who participated in the ED community for the first time during the midpandemic period.

**Table 1:** Descriptive statistics of comment threads (N=17,715) in the eating disorder community on the social media website Reddit.

Variable	Prepandemic threads	Midpandemic threads	Total
Threads, n (%)	8772 (49.5)	8943 (50.5)	17,715 (100)
Threads per day, mean (SD)	66.45 (16.70)	129.61 (43.91)	88.13 (41.74)
Users per thread^a^, mean (SD)	3.43 (3.58)	4.6 (6.33)	4.02 (5.19)
Comments per thread, mean (SD)	3.24 (5.20)	4.74 (9.24)	4.00 (7.55)
LIWC^b^ word count per thread, mean (SD)	204.03 (247.54)	311.76 (485.53)	258.41 (390.18)
LIWC dictionary words per thread, mean (SD)	89.83 (5.06)	90.51 (4.62)	90.17 (4.85)

^a^Users per thread includes the post author and all users that commented on the thread.

^b^LIWC: Linguistic Inquiry and Word Count.

The labels of the final topic model, exemplary words for topics and LIWC categories, and their unstandardized mean prevalence rates in threads before and after March 11, 2020, are shown in Table 2. Topics can be subsumed into broad categories such as ED symptom-related topics (*binge or restrict*, *purging*, *binge foods*, *low calorie foods*); weight, shape, and eating concerns (*weight loss or gain*, *body dysmorphia*, *exercise*, *appearance*, *meals*); mental health and treatment (*mental health*, *ED treatment*); everyday life (*domestic life*, *entertainment*, *drinks*); social aspects (*romantic relationships*, *social support*); EDs in community and society (*ED communities*, *EDs and society*); and expressions of emotions (*affect*).

**Figure 1:**
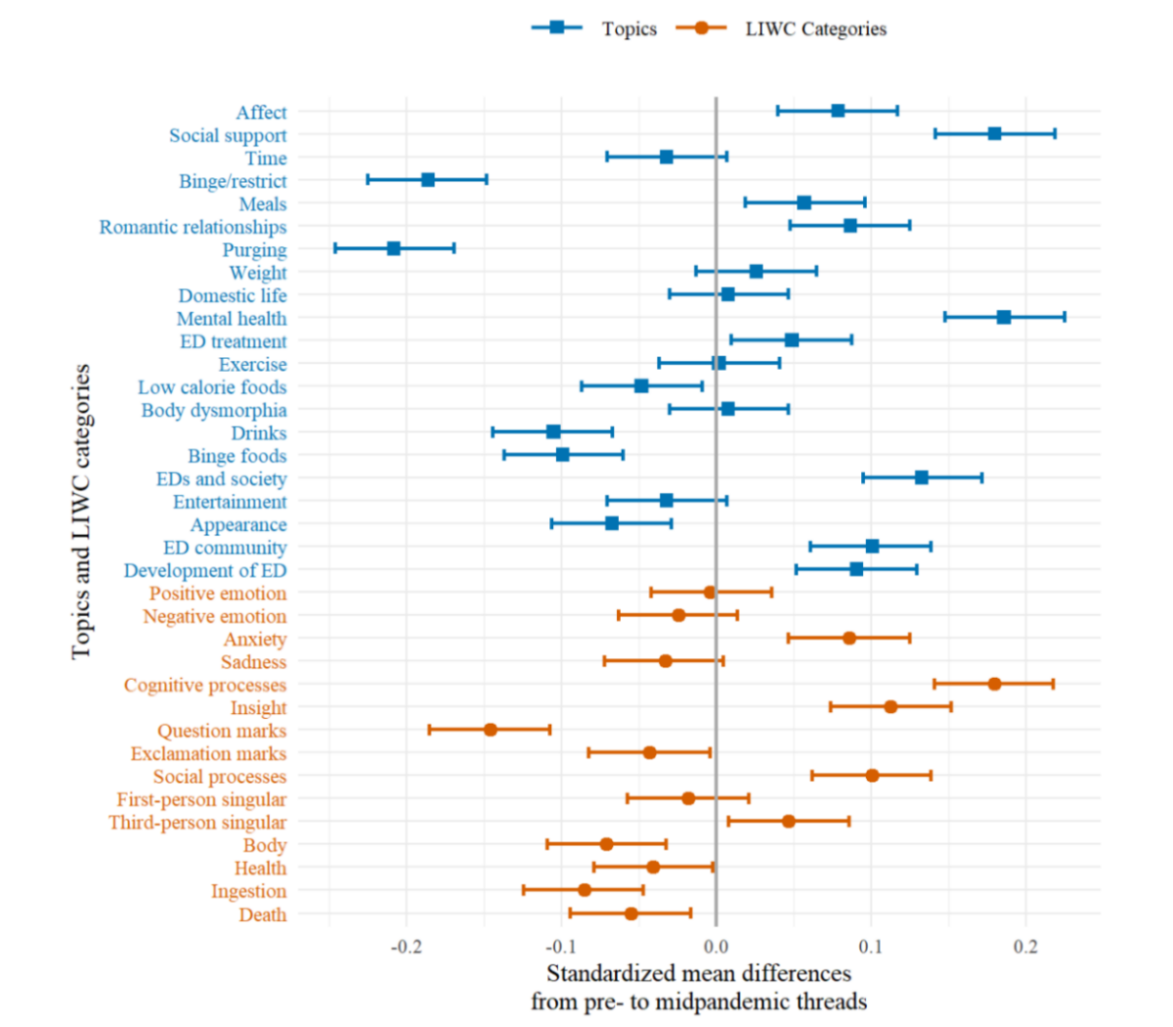
Standardized mean differences of the prevalence of topics and LIWC categories from pre- to midpandemic threads. ED: eating disorder; LIWC: Linguistic Inquiry and Word Count.

Overall, the most common topics were *affect*, *support*, *time*, and *binge or restrict*. The order of topics from most to least prevalent changes from pre- to midpandemic threads, with the topics *meals* and *romantic relationships* becoming more common than the topic *binge or restrict* as an example. SMDs of topics between pre- and midpandemic threads are shown in Figure 1. A total of 15 out of the 21 topics showed a significant change, that is, they had CIs that did not include zero. Significant increases in the first 2 months of the pandemic compared to the prepandemic time period were observed in the prevalence of the following nine topics: *affect* (SMD 0.079, 99% CI 0.040-0.117), *social support* (SMD 0.180, 99% CI 0.142-0.219), *meals* (SMD 0.057, 99% CI 0.019-0.096), *romantic relationships* (SMD 0.087, 99% CI 0.048-0.125), *mental health* (SMD 0.186, 99% CI 0.148-0.225), *ED treatment* (SMD 0.049, 99% CI 0.01-0.088), *EDs and society* (SMD 0.133, 99% CI 0.095-0.172), *ED community* (SMD 0.101, 99% CI 0.061-0.139), and *development of ED* (SMD 0.091, 99% CI 0.052-0.13). The prevalence of the following six topics decreased in midpandemic threads in relation to prepandemic threads: *binge or restrict* (SMD –0.186, 99% CI –0.225 to –0.148), *purging* (SMD –0.208, 99% CI –0.246 to –0.169), *low calorie foods* (SMD –0.048, 99% CI –0.087 to –0.009), *drinks* (SMD –0.105, 99% CI –0.144 to –0.067), *binge foods* (SMD –0.099, 99% CI –0.137 to –0.06), and *appearance* (SMD –0.067, 99% CI –0.106 to –0.029). Figures of daily mean prevalence rates of all topics between November 1, 2019, and May 20, 2020, can be found in Supplement S3 in Multimedia Appendix 1.

Out of the 13 LIWC categories, 9 showed a significant change, that is, they had a 99% CI excluding zero*.* Five LIWC categories, *anxiety* (SMD 0.086, 99% CI 0.047-0.125), *cognitive processes* (SMD 0.18, 99% CI 0.141-0.218), *insight* (SMD 0.113, 99% CI 0.074-0.152), *social processes* (SMD 0.101, 99% CI 0.062-0.139), and *third-person singular* (SMD 0.047, 99% CI 0.008-0.086), became more frequent in mid- compared to prepandemic threads. *Question marks* (SMD –0.146, 99% CI –0.185 to –0.107); *exclamation marks* (SMD –0.043, 99% CI –0.082 to –0.004); and words from the LIWC categories *body* (SMD –0.071, 99% CI –0.109 to –0.032), *health* (SMD –0.041, 99% CI –0.079 to –0.002), *ingestion* (SMD –0.085, 99% CI –0.124 to –0.047), and *death* (SMD –0.055, 99% CI –0.094 to –0.016) were used less frequently in threads after the onset of the COVID-19 pandemic. Figures of daily mean prevalence rates of all LIWC categories between November 1, 2019, and May 20, 2020, can be found in Supplement S4 in Multimedia Appendix 1.

**Table 2:** Names, exemplary words, and unstandardized prevalences of topics (n=21) and LIWC categories (n=15) in a corpus of comment threads from the ED community on the social media website Reddit.

Name		Exemplary words^a^	Prevalence in corpus^b^, mean (SD)	
			Prepandemic threads	Midpandemic threads
**Topics**				
	Affect	feel hate brain bad just like els	11.16 (5.33)	11.58 (5.49)
	Social support	hope happi deserv thank proud better strong	7.2 (5.67)	8.33 (6.69)
	Time	back month ve start ago sinc now	6.81 (4.12)	6.68 (4.09)
	Binge/restrict	bing fast tomorrow day restrict urg christma	6.91 (6.60)	5.76 (5.68)
	Meals	hungri eat meal food dinner hunger lunch	5.98 (5.33)	6.30 (5.87)
	Romantic relationships	friend tell boyfriend said ask told partner	5.64 (5.26)	6.12 (5.77)
	Purging	ive im throat cant ur vomit spit	6.49 (6.45)	5.22 (5.76)
	Weight loss/gain	gain scale pound lbs lose weight maintain	5.42 (7.55)	5.62 (7.62)
	Domestic life	home hous room kitchen money groceri car	4.21 (5.61)	4.26 (5.99)
	Mental health	disord anorexia behavior valid mental ed behaviour	3.80 (4.30)	4.66 (4.86)
	ED^c^ treatment	doctor hospit treatment appoint medic inpati therapist	3.81 (7.15)	4.16 (7.23)
	Exercise	burn sleep gym workout faint exercis dizzi	3.93 (5.87)	3.94 (6.13)
	Low calorie foods	veggi vegan salad veget soup carrot sauc	3.84 (8.43)	3.45 (7.90)
	Body dysmorphia	mirror attract photo skinnier compliment skinni thinner	3.7 (5.71)	3.74 (5.79)
	Drinks	drink coffe tea soda coke caffein sweeten	4.08 (9.60)	3.14 (8.16)
	Binge foods	cooki chocol peanut cake cream chip pizza	3.91 (7.56)	3.20 (6.81)
	EDs and society	peopl cultur judg societi shame agre opinion	3.07 (4.08)	3.66 (4.69)
	Entertainment	movi song hair scene mukbang video film	2.89 (6.68)	2.68 (6.21)
	Appearance	cloth wear hip boob jean waist shirt	2.95 (7.43)	2.47 (6.85)
	ED communities	sub reddit post subreddit pro account delet	2.15 (4.86)	2.69 (5.98)
	Development of ED	sister parent grade mom school teacher mum	2.04 (2.99)	2.32 (3.29)
**LIWC^d^** **categories**				
	Positive emotion	love, nice, sweet	3.41 (2.70)	3.40 (2.54)
	Negative emotion	hurt, ugly, nasty	3.64 (2.66)	3.57 (2.33)
	Anxiety	worried, fearful	0.58 (0.95)	0.67 (0.94)
	Sadness	crying, grief, sad	0.87 (1.27)	0.83 (1.10)
	Cognitive processes	cause, know, ought	13.45 (4.70)	14.26 (4.27)
	Insight	think, know	2.48 (1.92)	2.69 (1.74)
	Question marks	?	1.41 (3.55)	0.99 (1.99)
	Exclamation marks	!	1.06 (2.85)	0.94 (2.74)
	Social words	mate, talk, they	6.30 (4.57)	6.76 (4.49)
	First-person singular	I, me, mine	10.08 (4.07)	10.01 (3.78)
	Third-person singular	she, her, him	0.59 (1.45)	0.66 (1.51)
	Body	cheek, hands, spit	1.41 (2.10)	1.28 (1.70)
	Health	clinic, flu, pill	1.67 (2.13)	1.60 (1.73)
	Ingestion	dish, eat, pizza	4.18 (3.72)	3.88 (3.36)
	Death	bury, coffin, kill	0.12 (0.51)	0.10 (0.40)

^a^For topics, exemplary words are the most characteristic words as measured by the FREX metric [37]. For LIWC categories, exemplary words are taken from the LIWC manual [40].

^b^For topics, prevalence in corpus is measured as the percentage of each topic in the corpus. For LIWC categories, it represents the relative percentage of category words per thread.

^c^ED: eating disorder.

^d^LIWC: Linguistic Inquiry and Word Count.

## Discussion

### Principal Findings

This study is the first to explore discussions in one of the largest ED online communities on the social media website Reddit during the onset of the global COVID-19 pandemic. We used automated text analysis methods to investigate changes in language style and topics in comment threads around the time when COVID-19 was declared a global pandemic on March 11, 2020. We were able to identify 21 topics in comment threads using LDA, addressing a number of domains and behaviors of individuals who were actively contributing to the online ED community. They cover areas such as ED symptoms; weight, shape, and eating concerns; mental health and treatment; everyday social life; or the expression of emotions. Additionally, we investigated changes in language styles at the beginning of the global pandemic using a set of indicators covering affective, cognitive, social, and behavioral dimensions of language use.

As the two text analysis methods used in our study follow different approaches, bottom-up in the case of LDA and top-down in the case of LIWC, their combined results provide a more complete picture of discussion in the ED community than either method on its own. By providing unique insights into the online social media behaviors in this large community, our results complement and contrast with existing survey studies on the effects of the pandemic on individuals with EDs (eg, [12,13]) and a qualitative analysis of other ED communities on Reddit [28]. With this study, we followed the call to study the experiences of individuals with or at risk of EDs during the global COVID-19 pandemic, as they can aid in developing studies and interventions that address the needs of these vulnerable groups in future crises [14].

Although the focus of our study was not on individual users but rather on the community as a whole, our findings were still in line with another study that showed that individuals with EDs were more active on social media during the first months of the COVID-19 pandemic [11]. Recurring users, that is, users who were active in both time periods of our study, had higher participation rates in threads in the midpandemic period. Additionally, a high number of new users joined and participated in the ED community in the midpandemic period. However, the actual number of new individuals in the ED community is most likely lower, as many Reddit users abandon their existing accounts after some time and create new accounts.

Topics covering ED symptom–related discussion such as *binge or restrict*, *purging*, *low calorie foods*, or *binge foods* had lower prevalences in midpandemic than in prepandemic threads*.* This was supported by a decrease in the LIWC category *ingestion* after March 11, 2020, which suggests that the communication in the community focused less frequently on ED symptom–related discussions at the onset of the global COVID-19 pandemic. This pattern stands in contrast to recent surveys that found marked increases in self-reported ED symptoms during the COVID-19 pandemic [9,11-13,16]. However, a qualitative study of ED communities during this time also noted a reduction in ED symptoms in a small percentage of users [28].

At the onset of the COVID-19 pandemic, traditional and social media were focusing on reports about food shortages, weight gain during lockdowns, and home exercises. However, this media attention did not lead to an increase in discussion about topics related to weight, exercise, and body dysmorphia in the ED community, possibly because users already show a high level of preoccupation with these topics. It is striking that communicating about physical exercise has not become more common, as surveys with individuals with an ED indicate an increase in exercising during the pandemic [11]. However, we observed a significant decrease in the prevalence of the *appearance* topic, which could indicate that users worry less about how they look at this time, most likely because they are staying at home more where they will be seen by fewer people.

Mental health and treatment-related topics became more prevalent in the ED community after the beginning of the pandemic. In their discussions, users might have used social media to address their experiences and concerns with treatment disruptions or the transition of health care services to telehealth applications [9,11,13]. However, it could also reflect a genuine effort by users to learn about ED treatments or exchange views on recovery from EDs. A willingness among users of ED communities on Reddit to foster healthier habits and attempt recovery from EDs during the first months of the pandemic was also noted in another study [28]. The increase in discussions could have also been caused by users debating or inquiring about alternatives for their interrupted ED treatments such as self-management of recovery or anonymous helplines [9,17].

On a cognitive level of language, we found a rise in indicators that were associated with cognitive processing, that is, the LIWC categories *cognitive processes* and *insight*, in threads. Cognitive processing was previously found to be elevated in blogs advocating recovery from EDs (pro-recovery) compared to pro-ED blogs [39]. This was attributed to individuals in stages of recovery showing greater cognitive reflection or reappraisal of their condition in their blogs. In line with these findings, we also observed an increased language complexity through the decreased use of exclamation marks.

Two topics demonstrating higher levels of cognitive processing were also featured more frequently in midpandemic comment threads. The topic *EDs and society* subsumes discussions on how EDs and individuals with lived experience of EDs are perceived by society as indicated by words such as *culture*, *shame*, or *judge*. With the topic *development of ED*, users reflected on events or persons in their past that they believe to have influenced the onset and development of their ED behaviors and thoughts. These discussions required a higher level of abstraction and recollection from their participants.

Users’ social life seemed to play a bigger role in the community, as increases in the topic *romantic relationships*, the LIWC category *social words,* and in personal pronouns referring to another person (*third-person singular*) showed. Due to lockdowns, stay-at-home orders, and similar measures, users might be spending more time at home with their partners and families. This can be challenging for individuals with EDs, as they might feel pressured to follow a diet set by others or stressed because they are hiding their ED behaviors from others [11]. Users might be venting about social interactions or conflicts with others in their household using words from the aforementioned categories and topics. Shared meals represent another source for potential conflicts, as others can observe what and how much the individual with an ED is eating, which could have led to the increase in discussions about *meals* [11]. The amount of social references differentiated between pro-recovery and pro-ED content in a previous study, with less social references found in pro-ED texts and more social activity being associated with recovery [39]. This is attributed to a withdrawal from others or avoidance of connections with others typical for EDs. The higher frequency of social references in our study indicates that users are either having more real-world interactions about which they reflect in the online community or, alternatively, that more of their social life is happening in the community because real-life social relationships were restricted, both supporting a positive trend away from ED-typical social withdrawal.

The rise in frequency of anxiety-related words could be an indicator for elevated health anxiety and worries about contracting SARS-CoV-2 [9,10]. Indeed, health anxiety has emerged across a number of mental health communities including the ED community investigated in this study in the wake of the pandemic [41]. Additionally, anxiety about COVID-19 was related to higher ED pathology in one study [42]. In this context, it is surprising that we found a decrease in prevalence of the LIWC categories assessing *health* and *death*, as these encompass words that could be used to discuss COVID-19, its treatment, and sequelae.

Although it might appear contradictory to observe a rise in the topic *affect* without a corresponding increase in neither the *positive emotions* nor *negative emotions* categories, this discrepancy can be explained by the composition of each category. The *affect* topic captured words representing emotions, such as shame, guilt, or anger, which are, however, featured to a much lower degree in the *positive emotions* or *negative emotions* dictionaries.

The increase in prevalence of anxiety words reflects the expression of fears associated with the serious consequences of COVID-19; however, it also points to a rise in emotional awareness and disclosure that would be more typical of recovery texts [39]. A stronger inhibition of negative emotions resulting in lower expression of negative emotion words would be expected in individuals affected by EDs rather than in individuals currently recovering from an ED. However, we did not observe any significant reductions in words from the LIWC category *sadness* in midpandemic threads.

In sum, our results illustrate pronounced changes in the online community that suggest a perspective shift in the discussions in the ED community from a narrow focus on ED symptoms and potentially harmful weight loss methods toward a broader perspective with increased attention to mental health, social resources, and treatments for EDs. This shift is characterized by heightened cognitive processing, more social references, less inhibition of negative emotions, and discussions focused less on ED symptoms and more on mental health and treatment. It is important to note here that the rules set by the community’s moderators claim to not explicitly identify as either being pro-recovery or pro-ED, as the community would offer support to all and would encourage or promote neither ED behaviors nor recovery from EDs (quote paraphrased to protect the anonymity of the ED community). Although some social media platforms have distinct pro-recovery and pro-ED communities, which show seemingly little interaction between their members [43], other online ED communities feature pro-recovery and pro-ED content alongside each other [44]. It is therefore possible that the communication pattern observed in an ED community might move toward the pro-recovery end of a theoretical spectrum between pro-ED and pro-recovery. It is yet unclear whether this is a sustained change or a temporary phenomenon pushed by the disruptive situation of the recent pandemic. We also cannot deduce from these results whether they will lead to many users attempting or achieving recovery from EDs, as this can be a protracted and difficult process that is often accompanied by relapses. Remaining active in online ED communities could make achieving recovery even more difficult [44].

Other salient results from this study concern factors such as social support and the ED community. Expressions of social support became more frequent at the beginning of the pandemic. However, social support in online ED communities can be a double-edged sword, as many users report that they value the communities for the support they provide [26], while researchers suggest that support is often tied to following group norms that foster potentially harmful ED behaviors and thoughts [20]. Therefore, supportive words might be extended to users directly affected by the pandemic, to those seeking help with their condition, and potentially to those wanting to lose weight using potentially harmful methods. The topic *ED community* contains references to the ED community and Reddit in general. It could represent a kind of meta-discussion about the community and its place among other communities on Reddit with the topic. Its significant increase might reflect a kind of growth in awareness of the community and how it can support its users. These potential consequences of the pandemic on the structure of social media warrant further exploration in future research.

### Limitations

This study is limited by the fact that we do not know what the relation between discussions in an ED community and real-life behaviors is. Although we found decreases in ED symptom–related discussions and increases in discussions about social and mental health issues, we do not know whether these changes are accompanied by reductions in ED behaviors and increased help-seeking or treatment uptake. However, the shift in discussion points to the community itself becoming a place that is more open to treatment and recovery from EDs.

A second limitation is that the ED community and Reddit as a whole can be used anonymously, precluding us from making inferences on the demographics or ED impairment of users in the community. Additionally, we cannot draw conclusions on changes due to the pandemic for particular users or user groups, as we were interested in changes in the community as a whole and thus estimated prevalences of language style and topics at the level of threads. As our analysis methods treat these threads, which combine a post and comments from different users as bags of words, we were unable to trace back the prevalence of topics and LIWC categories or their changes to specific users or groups of users. Although it is possible that the same users went from discussing ED symptoms to asking about mental health and treatment, it could also have been entirely new users that changed the discussion.

A third limitation concerns the time period under study. We observed the online ED community in the first few months of the pandemic, and it is yet unclear whether the observed changes will endure over time. Additionally, we cannot ascertain whether the changes we observed are fully due to the COVID-19 pandemic or whether seasonal effects also play a role. Plots of mean daily prevalence rates of topics and LIWC categories over the whole time span of our study in Supplement S3 and S4 in Multimedia Appendix 1 can give some indication whether effects are due to short spikes or more enduring trends.

### Conclusions

The COVID-19 pandemic and its accompanying public health measures have disrupted the everyday life of people worldwide, possibly impacting their psychological well-being and coping strategies. Clinical researchers are understandably concerned about how these changes affect individuals with mental disorders. We present in this study a snapshot of changes in an online ED community during the first few months of the pandemic. As such, we aim to contribute to the growing area of research on the experiences of individuals affected by disordered eating during this time. Our specific contribution lies in categorizing the natural discussions occurring in the ED community on Reddit into content and language style categories and uncovering how discussions changed at the beginning of the global pandemic. The presented results suggest that reaching out to users of online ED communities and recruiting them for treatment interventions might be especially effective at this time as need, openness, and interest for mental health treatment increases. Additionally, the language in discussions changed in a way that suggests a move toward a stronger focus on recovery and mental health treatment in the community. The changes we observed reflect issues users were experiencing in real life during the first wave of the COVID-19 pandemic. Understanding these issues can aid in developing interventions that can mitigate the consequences of future waves of the COVID-19 pandemic or other similar disease outbreaks in the future for individuals with EDs.

## Appendix

Multimedia Appendix 1 Supplementary material.

## Abbreviations

API: application programming interface
ED: eating disorder
LDA: latent Dirichlet allocation
LIWC: Linguistic Inquiry and Word Count
SMD: standardized mean difference
