# Supplementary material for: Changes in Language Style and Topics in an Online Eating Disorder Community at the Beginning of the COVID-19 Pandemic: Observational Study
Source: J Med Internet Res. 2021 Jul 8;23(7):e28346. doi: 10.2196/28346 (PMC8274670; doi:10.2196/28346)

Supplement S1. Exclusivity and semantic coherence of models with K = 3, 6, 9, 12…30 topics. The numbers next to the dot indicate the number of topics, *K*, in the model.


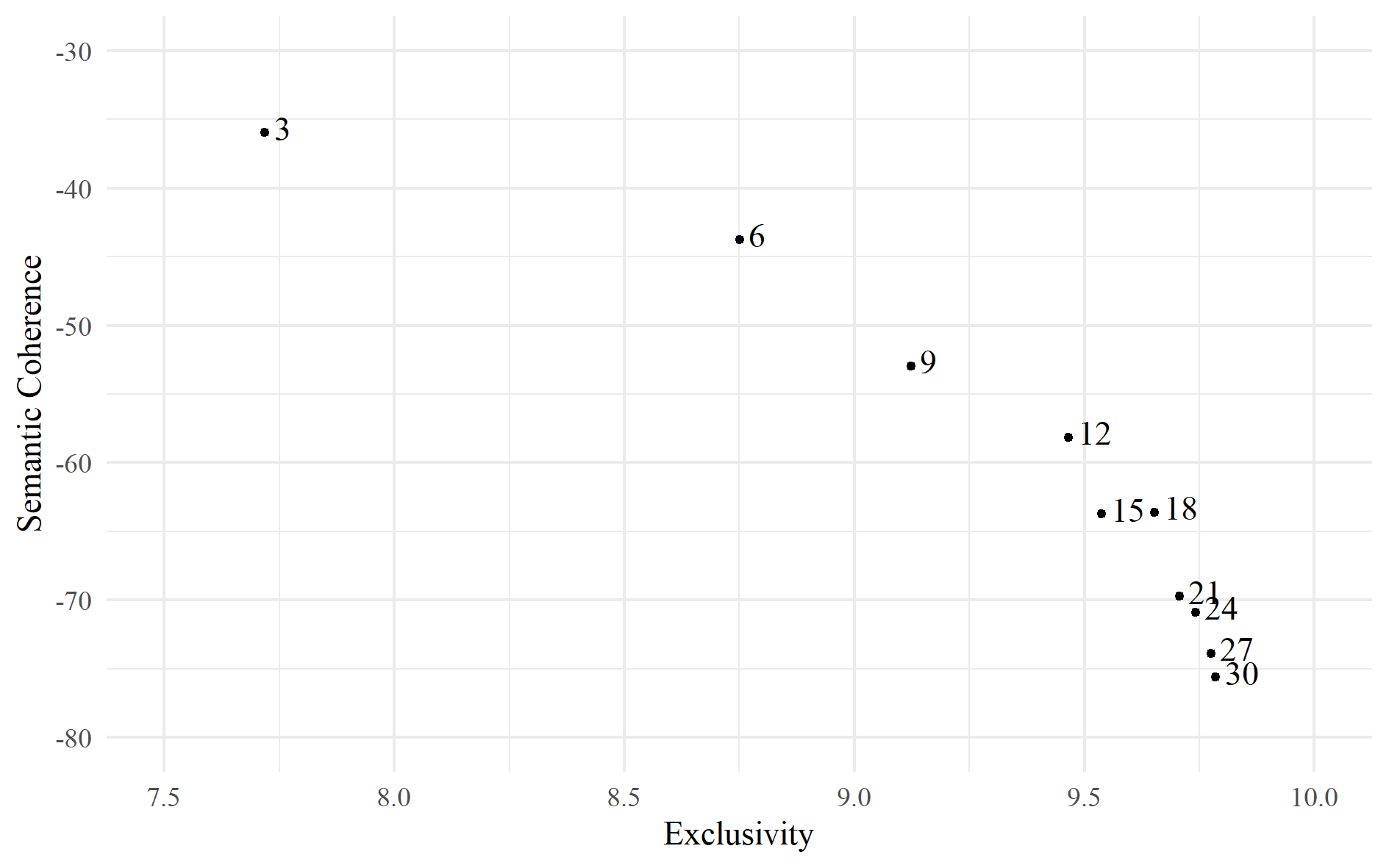


Supplement S2. Exclusivity and semantic coherence of models with K = 9-21 topics. The numbers next to the dot indicate the number of topics, *K*, in the model.


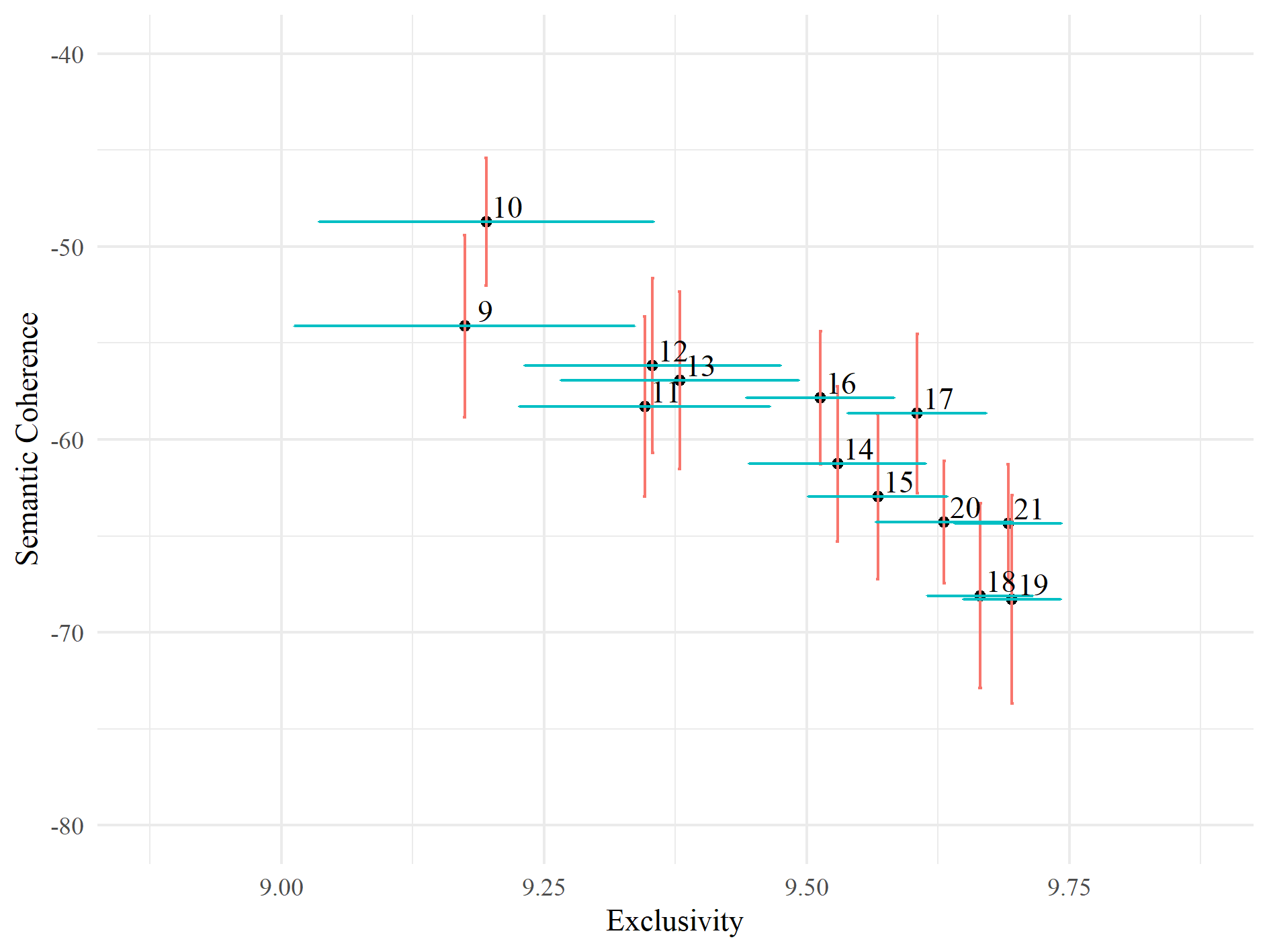


Supplement S3. Daily mean relative percentages of LIWC categories in N = 17,715 comment threads in an ED community on Reddit. The horizontal red line marks the date 11^th^ May 2020, chosen as the starting date for the mid-pandemic period in our study.


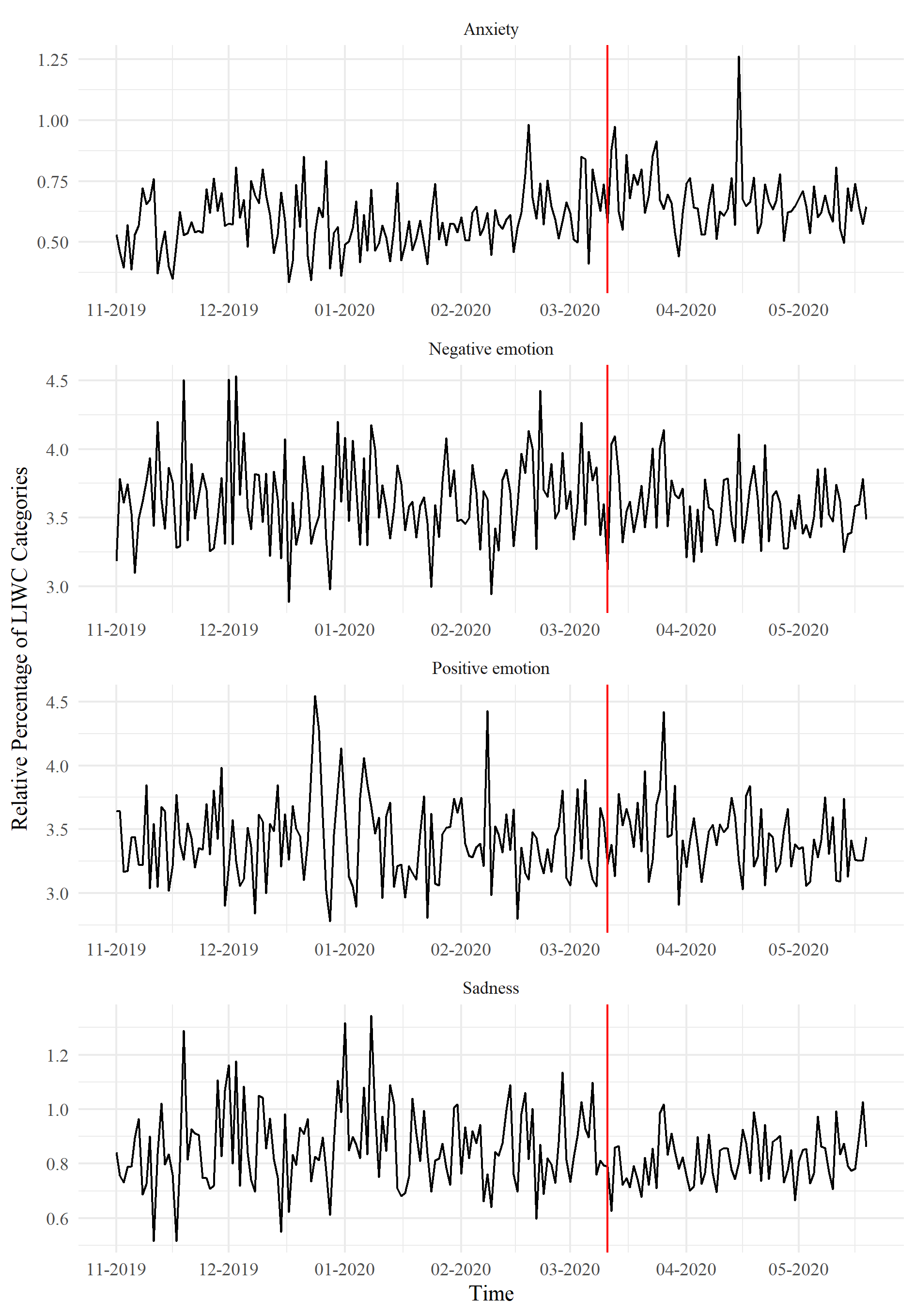


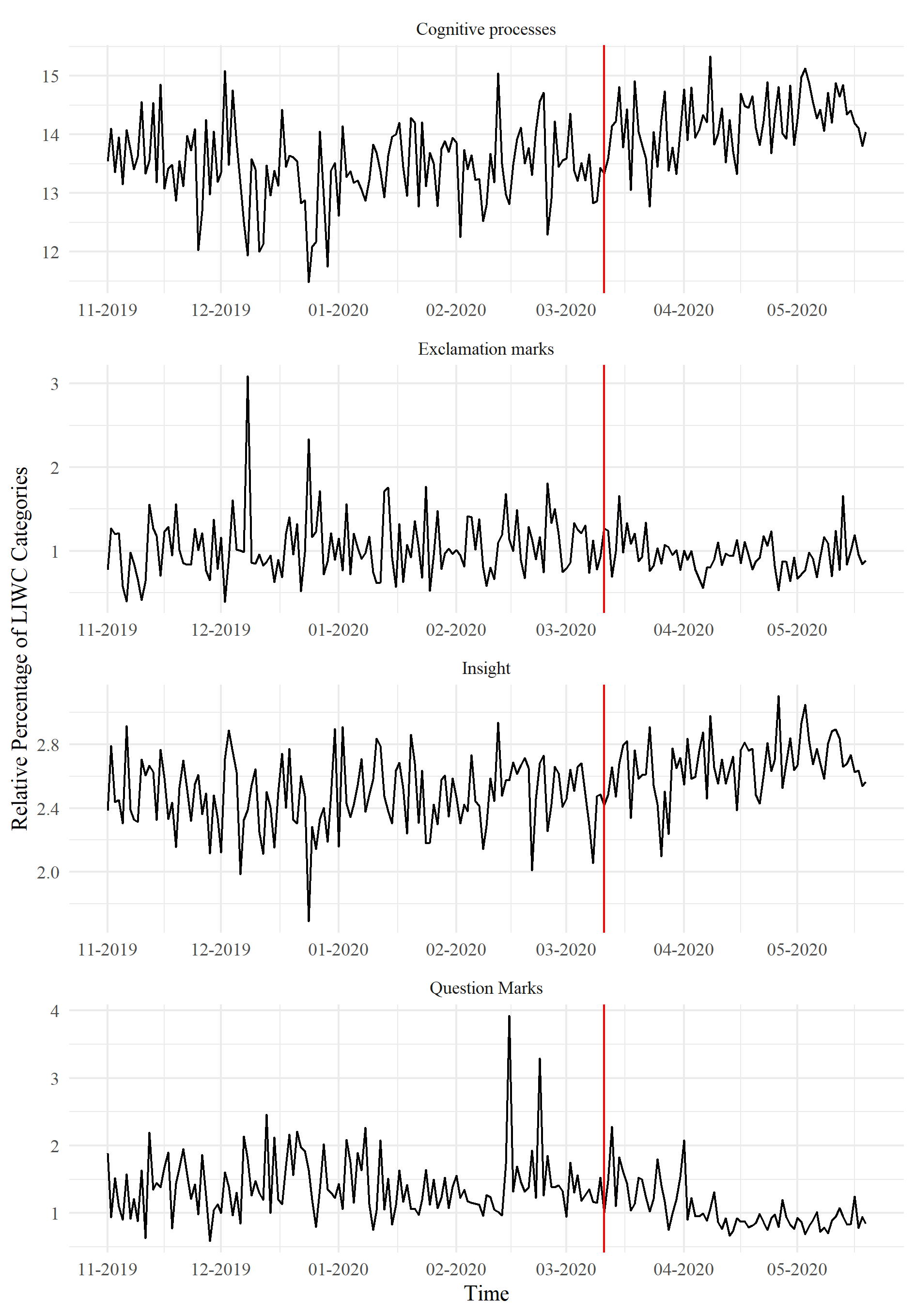


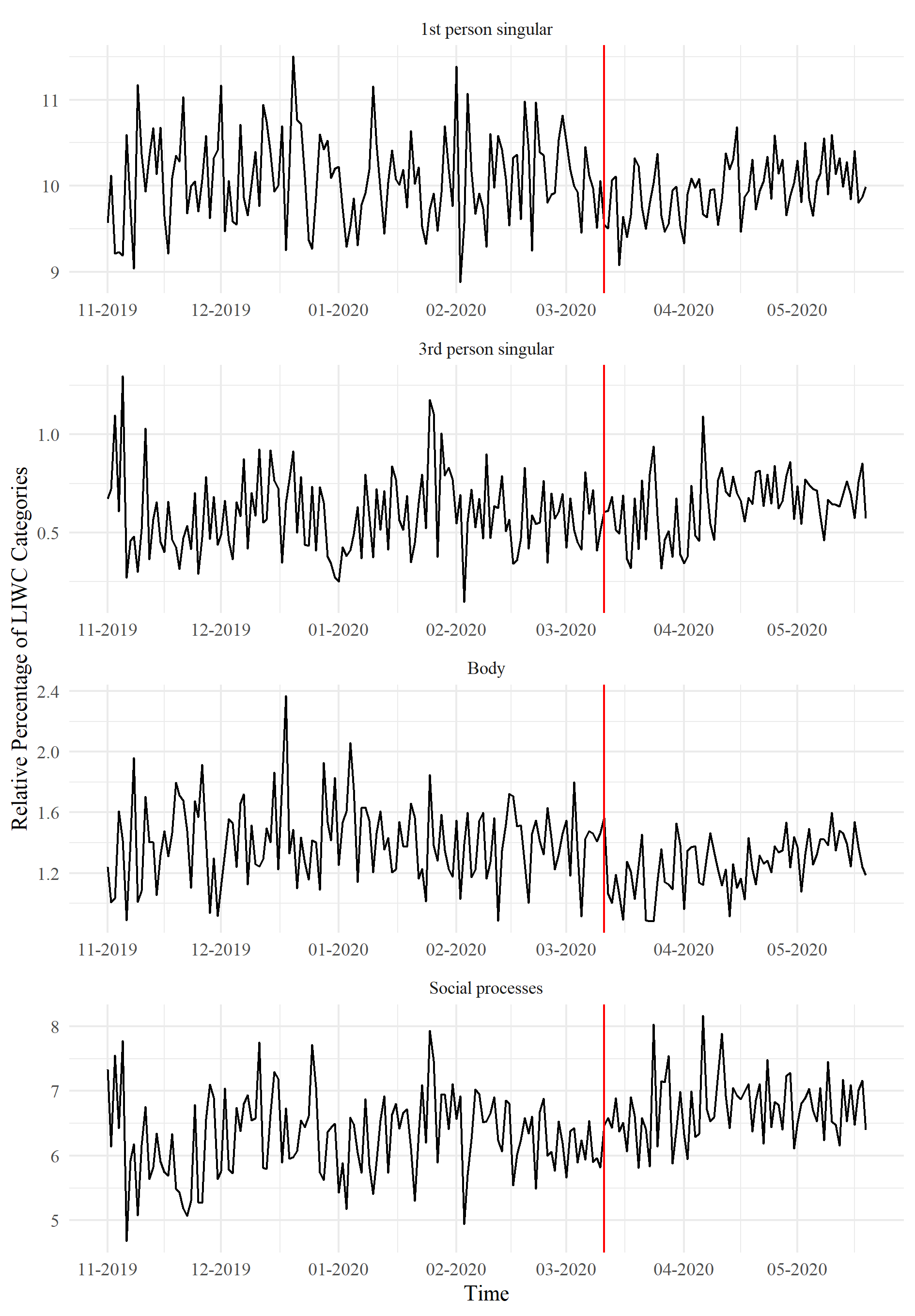


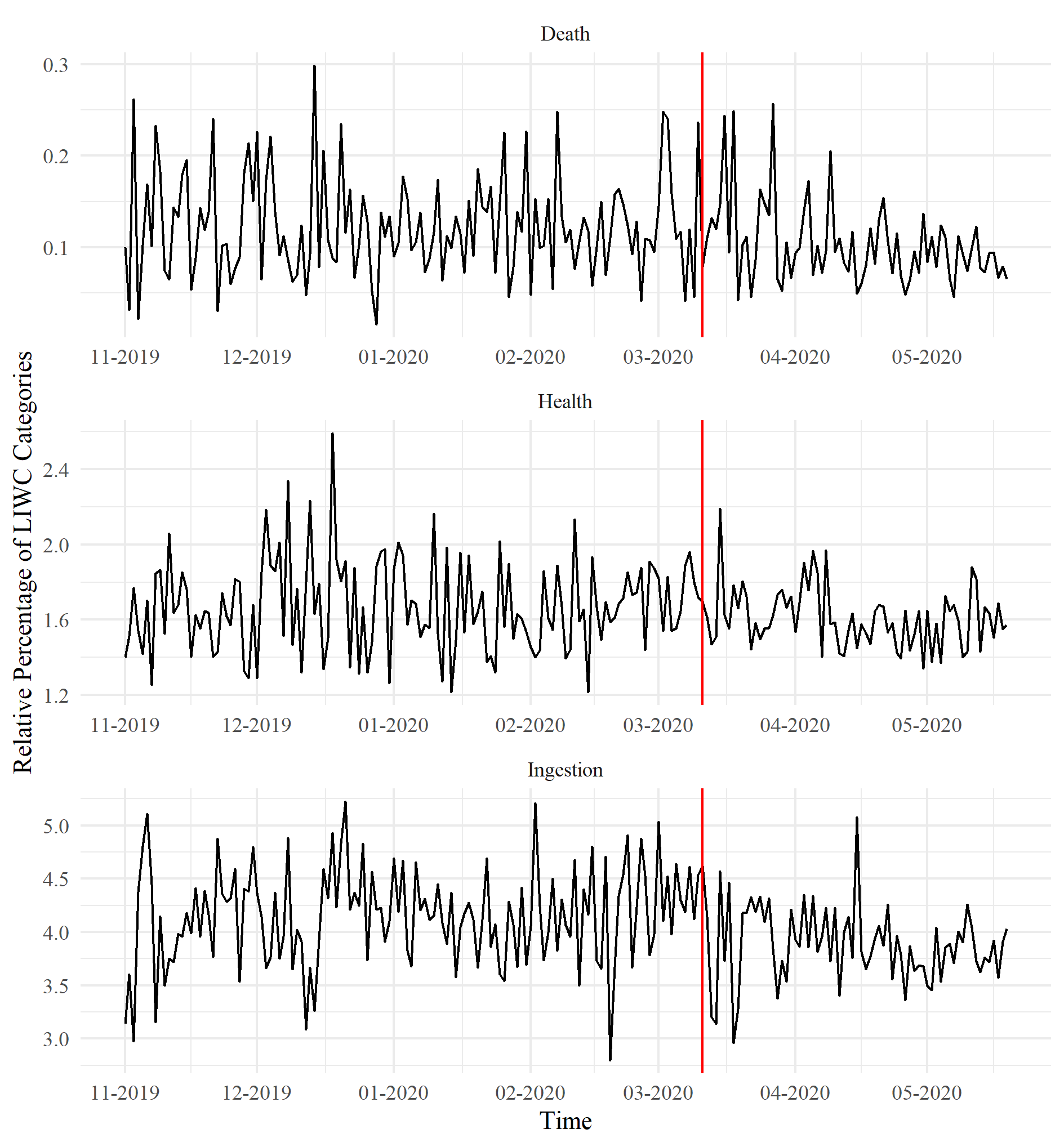


Supplement S4. Daily mean relative percentages of topics in N = 17,715 comment threads in an ED community on Reddit The horizontal red line marks the date 11^th^ May 2020, chosen as the starting date for the mid-pandemic period in our study.


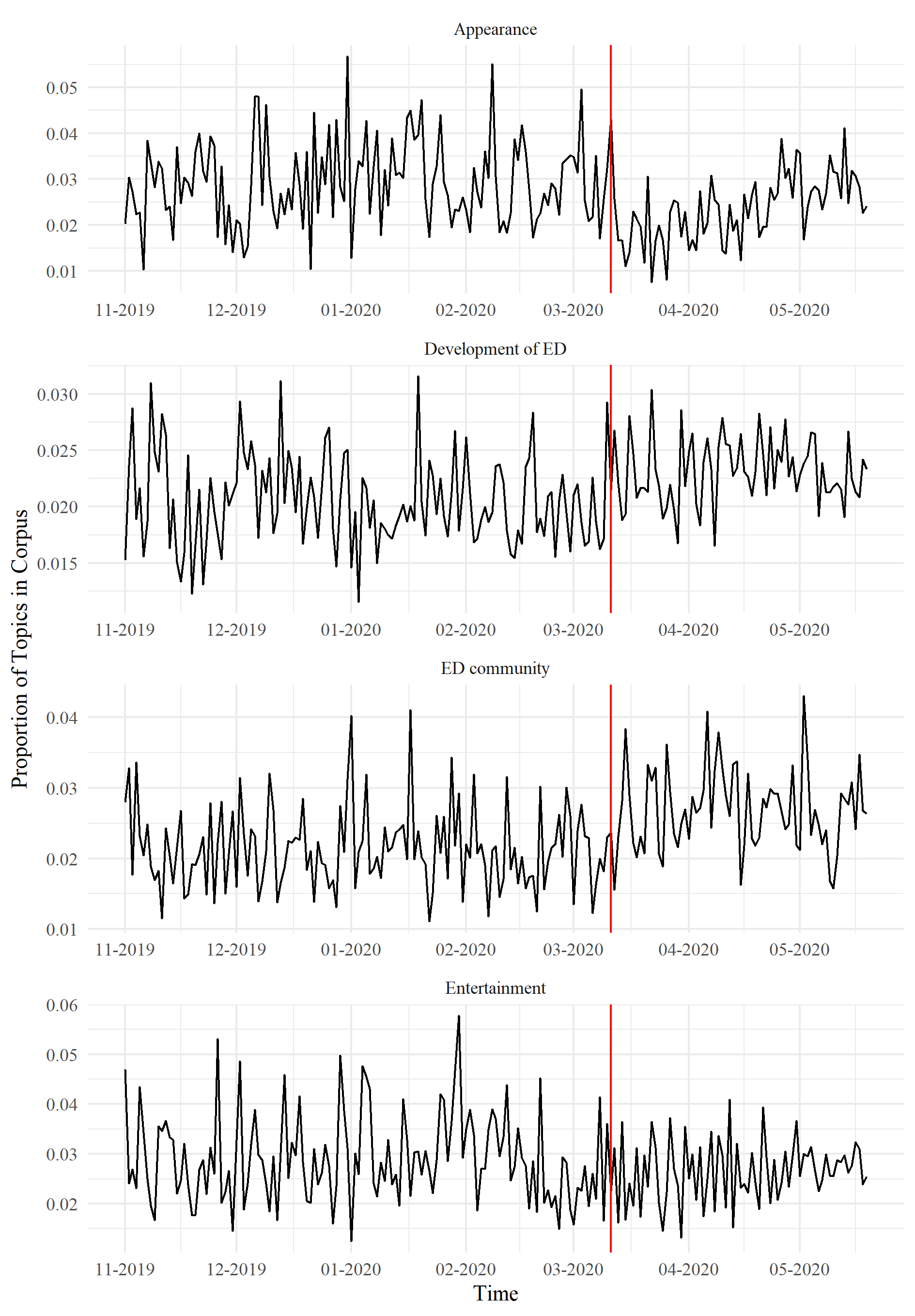


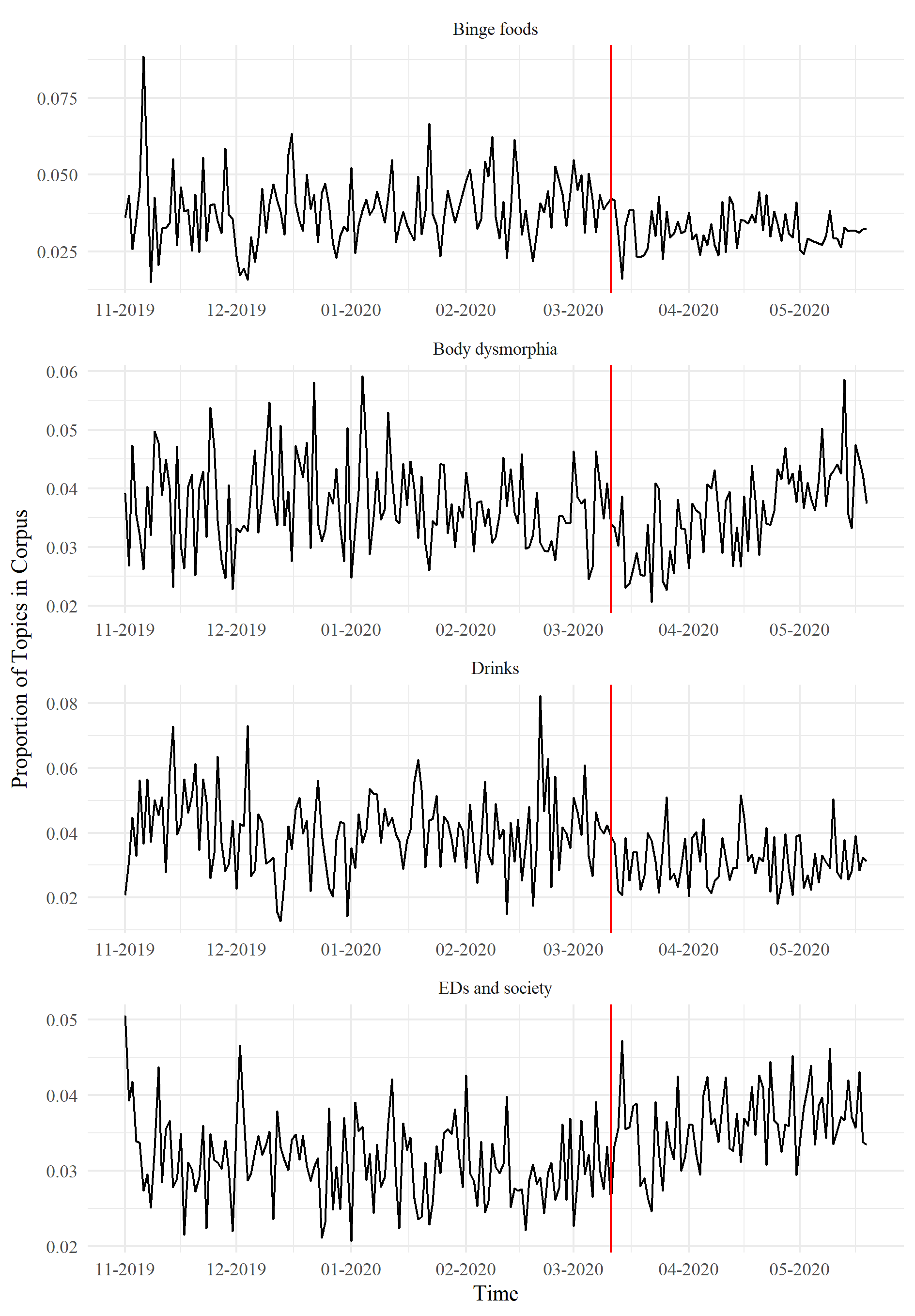


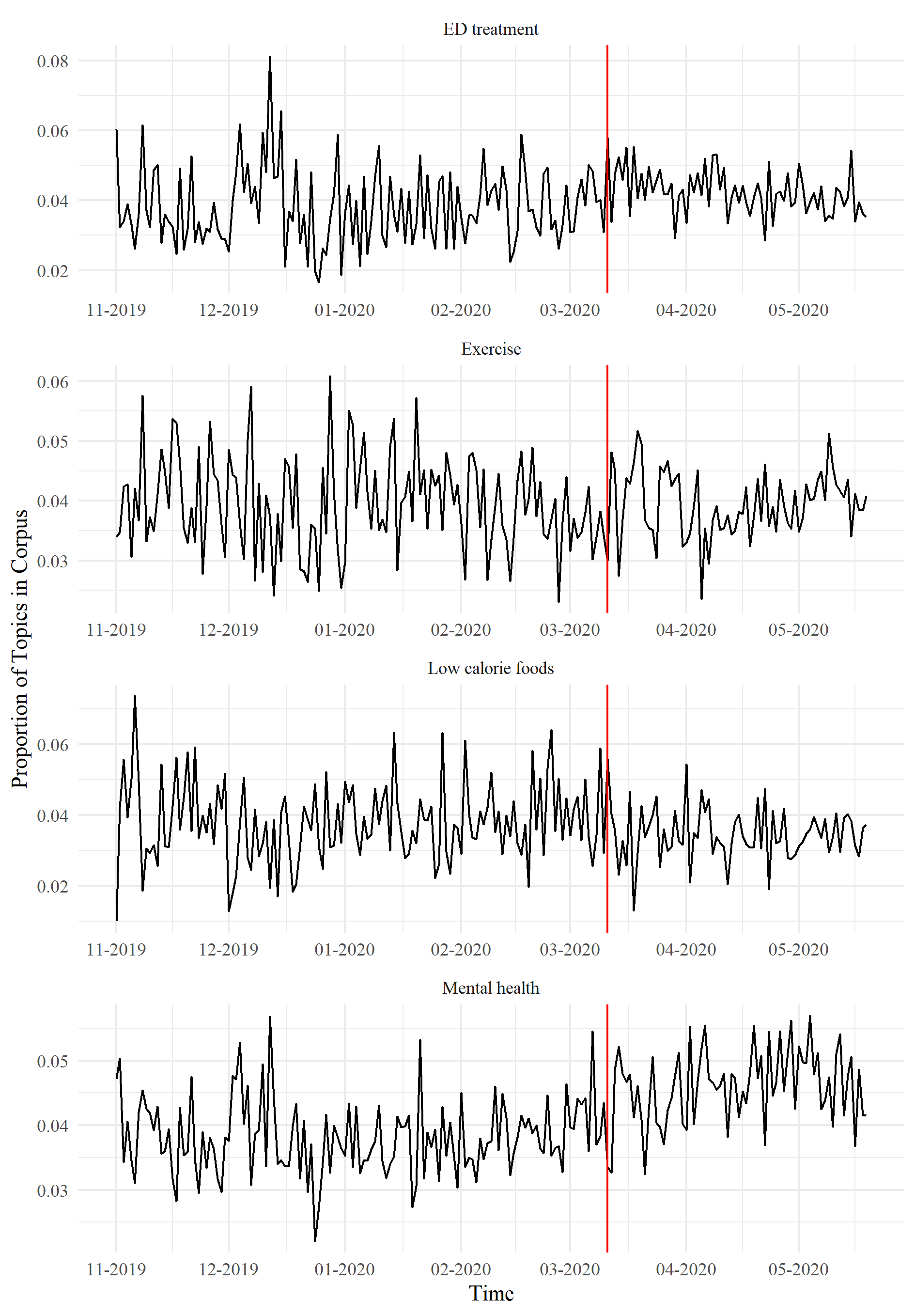


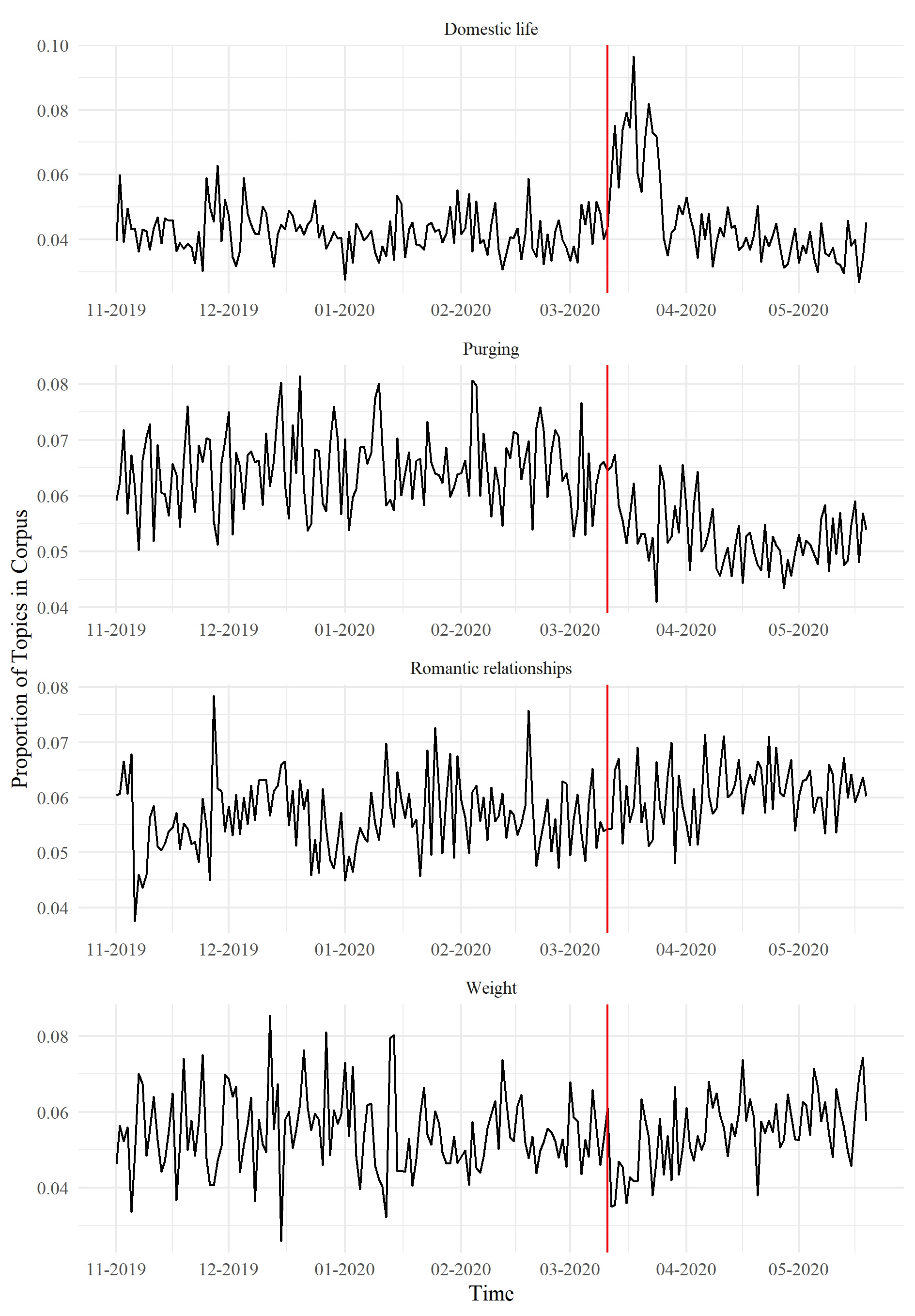


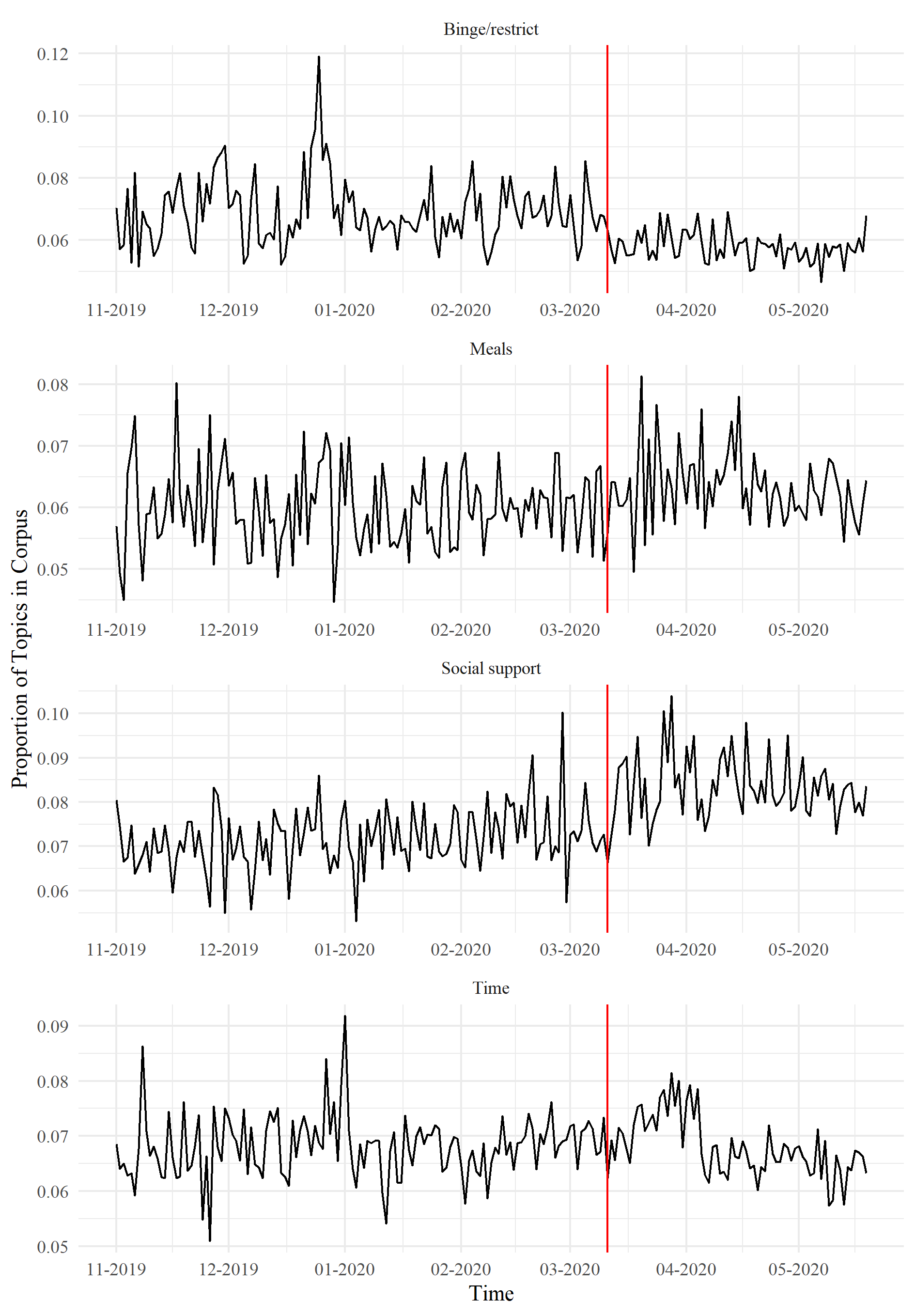


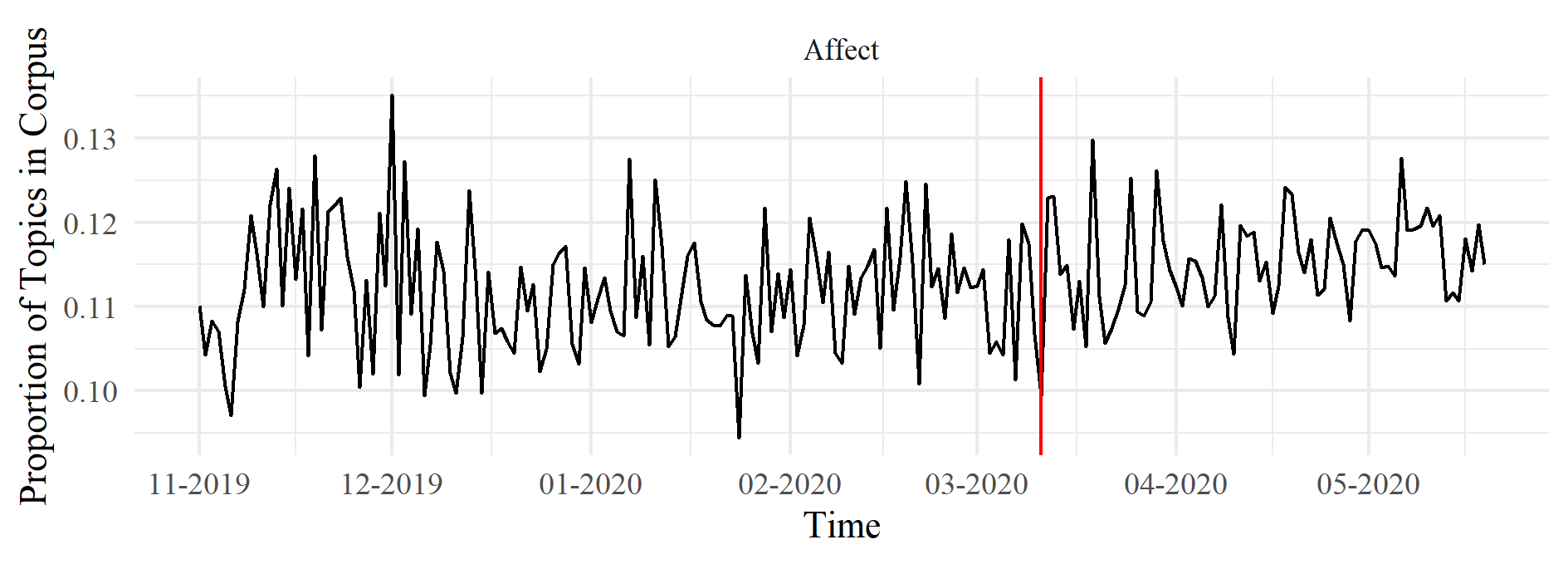

Supplement: Multimedia Appendix 1 [file jmir_v23i7e28346_app1.docx]
